# YAP/TAZ: Key Players for Rheumatoid Arthritis Severity by Driving Fibroblast Like Synoviocytes Phenotype and Fibro-Inflammatory Response

**DOI:** 10.3389/fimmu.2021.791907

**Published:** 2021-12-09

**Authors:** Robin Caire, Estelle Audoux, Guillaume Courbon, Eva Michaud, Claudie Petit, Elisa Dalix, Marwa Chafchafi, Mireille Thomas, Arnaud Vanden-Bossche, Laurent Navarro, Marie-Thérèse Linossier, Sylvie Peyroche, Alain Guignandon, Laurence Vico, Stephane Paul, Hubert Marotte

**Affiliations:** ^1^ INSERM, U1059-SAINBIOSE, Université de Lyon, Saint-Etienne, France; ^2^ CIRI (Centre International de Recherche en Infectiologie), Equipe GIMAP (Team 15), INSERM, U1111, CNRS, ENS, UCBL1, Université Jean Monnet, Université de Lyon, Saint-Etienne, France; ^3^ INSERM, U1059-SAINBIOSE, Mines Saint-Etienne, Université de Lyon, Saint-Etienne, France; ^4^ CIC INSERM, 1408, Université de Lyon, Saint-Etienne, France; ^5^ Department of Rheumatology, Hôpital Nord, University Hospital Saint-Etienne, Saint-Etienne, France

**Keywords:** YAP, mechanotransduction, inflammation, rheumatoid arthritis, inflammatory bowel disease

## Abstract

**Objective:**

The role of YAP/TAZ, two transcriptional co-activators involved in several cancers, was investigated in rheumatoid arthritis (RA).

**Methods:**

Fibroblast like synoviocytes (FLS) from patients with RA or osteoarthritis were cultured in 2D or into 3D synovial organoids. Arthritis rat model (n=28) and colitis mouse model (n=21) were used. YAP/TAZ transcriptional activity was inhibited by verteporfin (VP). Multiple techniques were used to assess gene and/or protein expression and/or localization, cell phenotype (invasion, proliferation, apoptosis), bone erosion, and synovial stiffness.

**Results:**

YAP/TAZ were transcriptionally active in arthritis (19-fold increase for CTGF expression, a YAP target gene, in RA *vs.* OA organoids; p<0.05). Stiff support of culture or pro-inflammatory cytokines further enhanced YAP/TAZ transcriptional activity in RA FLS. Inhibiting YAP/TAZ transcriptional activity with VP restored a common phenotype in RA FLS with a decrease in apoptosis resistance, proliferation, invasion, and inflammatory response. Consequently, VP blunted hyperplasic lining layer formation in RA synovial organoids. *In vivo*, VP treatment strongly reduced arthritis severity (mean arthritic index at 3.1 in arthritic group *vs.* 2.0 in VP treated group; p<0.01) by restoring synovial homeostasis and decreasing systemic inflammation. YAP/TAZ transcriptional activity also enhanced synovial membrane stiffening *in vivo*, thus creating a vicious loop with the maintenance of YAP/TAZ activation over time in FLS. YAP/TAZ inhibition was also effective in another inflammatory model of mouse colitis.

**Conclusion:**

Our work reveals that YAP/TAZ were critical factors during arthritis. Thus, their transcriptional inhibition could be relevant to treat inflammatory related diseases.

## Introduction

Yes-associated protein (YAP) and transcriptional co-activator with PDZ-binding motif (TAZ) are two transcriptional co-activators sharing strong structure similarities (1). They are activated in several cancer cells (2, 3). Upon specific *stimuli*, YAP/TAZ are translocated to the nucleus to induce transcription by interacting mainly with transcriptional enhanced associate domains (TEAD) (4). YAP/TAZ are partly regulated by the Hippo pathway, which leads to their retention in the cytoplasm (5). YAP/TAZ were first linked to tissue size homeostasis as they promote overgrowth when hyperactivated (6). In chronic inflammatory diseases, tissue resident cells could acquire tumor-like features and participate in inflammation development and tissue destruction (7–9). There are some emerging evidence that YAP transcriptional activity could promote chronic inflammatory diseases, especially in inflammatory bowel disease (IBD) (10–13).

Rheumatoid arthritis (RA) is the most common immune disorder characterized by joint inflammation and destruction (14). In RA, fibroblast-like synoviocytes (FLS), resident cells of the synovial tissue, acquire an aggressive phenotype including hyperproliferation, apoptosis resistance and invasion ability [partly linked to the secretion of matrix metalloproteinases (MMPs) such as MMP-2 and MMP-13 (15, 16)] persisting even after the inflammation has been suppressed (7). FLS are also key actors in initiating and maintaining the recruitment of immune cells (17, 18). The mechanisms involved in RA FLS phenotype are still unclear, with some contributions from c-Jun, a member of the activator protein 1 (AP-1) (19), nuclear factor κB (NF-κB) pathway (20), p53 or B-cell lymphoma 2 (Bcl-2) family members involved in apoptosis regulation (20, 21), and/or epigenetic alterations (22). Recently, epigenetic modifications of the Hippo pathway were also reported in RA FLS (23). Inhibition of YAP activity with verteporfin (VP), which blocks the binding between YAP/TAZ and TEAD (24), decreased RA FLS invasion and MMP-13 expression thus improving arthritis in mice (23). In addition to the involvement of YAP/TAZ in joint resident cells, TAZ was also described to increase the balance between IL-17 producing CD4+ T helper lymphocyte (Th17) and T regulatory lymphocyte (Tregs), stimulating the transcription of retinoic acid receptor-related orphan receptor-γ (RORγT) and leading to an increase of Th17 differentiation (25), which are the main source of IL-17. In RA, Th17 cells are major pro-inflammatory actors, and consequently, inhibition of YAP/TAZ could prevent systemic inflammation during arthritis.

YAP/TAZ are also major mechanotransduction actors converting cell mechanical *stimuli* (such as stiffening of the surrounding environment) into transcriptional responses for cell phenotype adaptation, independently of the Hippo pathway (26, 27). Interestingly, inflammation is commonly associated with increased cellular tension, through actin stress fibers (SF) formation (28). Inflammation can also trigger stiffening process in tissues through extracellular matrix (ECM) remodeling including stiff ECM component synthesis such as tenascin-C and periostin, which are two stiffening markers (29, 30). Furthermore, chronic inflammation has been linked to aberrant mechanotransduction responses that activate YAP/TAZ signaling and promote aberrant cell phenotype (29). However, such mechanisms remained unexplored in arthritis.

Thus, despite the recent evidence of YAP involvement in arthritis, several critical aspects are still lacking. First, YAP activity in RA FLS *in vitro* or *in vivo* was not highlighted. Second, it is still unknown how YAP activity may be modulated in FLS during arthritis. Third, in addition to the role of YAP in FLS invasion, the role of YAP in several other arthritis mechanisms such as FLS survival, global pro-inflammatory response, and synovial tissue remodeling is still to be determined. Here, we demonstrated strong YAP/TAZ transcriptional activity in RA FLS, enhanced by inflammation and mechanotransduction events that, in turn, regulated critical cellular responses involved in RA.

## Material and Methods

### Cell Culture

RA FLS were obtained during surgery procedure as previously described (18). All RA patients gave a written consent after oral information (IRB # 2014-A01688-39). FLS were cultured in Dulbecco’s modified Eagle’s medium (DMEM, Sigma, St. Louis, MO, US) with 10% fetal bovine serum (FBS), 1% glutamine and 2% penicillin and on classic support substrate (2 GPa) until their use for experiments. For HEK293 cells, the same culture medium was used without glutamine but with non-essential amino acid solution at 1%. HEK293 YAP^-/-^ were generated using specific CRISPR cas-9 and homology direct repair plasmid targeting YAP sequence (Santa Cruz Biotechnology, Dallas, TX, US), CRISPR clones generation was done following manufacturer instructions, and validated by western blot. For soft substrate culture (2 kPa), well plates (Cell guidance system, Cambridge, UK) or dishes (ExCellness, Lausanne, Switzerland) were used. Plates were coated with fibronectin (1:100, Sigma) for 2 hours at 37°C. TNF at 10 ng/ml and IL-17 at 50 ng/ml (R&D system, Minneapolis, MN, US) were used (except when specified in the Figures legend). Verteporfin (VP, Sigma) was used at 10 µM. For all VP experiments, cells were kept in relative darkness with blue light during manutention avoiding any aspecific activation of VP (due to its photosensitivity). For all experiments (except when specified) FLS were plated at low cell density (2,000 cells/cm^2^). HEK293 were grown on classic substrates coated with fibronectin at 100,000 cells/cm^2^.

### siRNA Transfection Technique

RA FLS were cultivated in classic medium antibiotic free for 24 hours and transfected at 80% confluency, before transfection FLS were rinsed using siRNA transfection medium (Santa Cruz Biotechnology, Dallas, TX, USA). RA FLS were transfected by adding YAP siRNA or control siRNA (Santa Cruz Biotechnology) at 7.5 µg/ml in siRNA transfection reagent (Santa Cruz Biotechnology; diluted 6/100 in transfection medium) for 6 hours at 37°C in a CO_2_ incubator. DMEM containing 20% FBS and 2% PS was added to the wells overnight (achieving a ½ dilution of the added medium). Medium was then replaced with or without TNF and IL-17 for 48 hours before extraction. On soft substrate, RA FLS siRNA transfection was noneffective due to bovine fetal serum retention in the gel.

### Western Blot

Protein extraction was performed with Allprep RNA/protein kit (Qiagen Inc, Hilden, Germany). Proteins (5 µg) were denatured and separated for 2 hours at 90 Volts before being transferred onto PVDF membranes (Thermo Fisher Scientific). The membrane was blocked and incubated with primary antibody overnight at 4°C. Then membrane was incubated with a horseradish peroxidase-conjugated secondary antibody (1:5000; Thermo Fisher Scientific, 31460) for 1 hour at RT. Immunoreactive protein bands were visualized with Clarity™ Western ECL Substrate (BioRad, Hercules, CA, US). Blots were stripped using a mild antibody stripping solution (200 mM glycine, 3.5 mM SDS, 1% Tween 20) and reprobed. Western blot (WB) was performed using the following primary antibodies diluted at 1:1,000: YAP/TAZ (8418, Cell Signaling Technology, Leiden, The Netherlands), CYR61 (14479, Cell Signaling Technology) MMP-13 (ab39012, Abcam, Cambridge, UK), NF-κB p65 (8242, Cell Signaling Technology), phospho NF-κB p65 ser536 (3031, Cell Signaling Technology) and 1:5,000: β-Actin (4970, Cell Signaling Technology).

### LDH Cytotoxicity Assay

RA FLS with density at 50,000 cells/well in 100 µL of medium were plated in triplicate wells in a 96-well tissue culture plate. A complete medium control without cells were included to determine LDH background activity, additional cells were plated in triplicate wells for measurement of spontaneous LDH activity control (medium) and maximum LDH activity controls (1X lysis buffer). The cells were treated with VP at different concentrations for 24 hours at 37°C, 5% CO_2_ and then the released of LDH in the supernatants was measuring using an LDH cytotoxicity assay kit (Thermo Fischer Scientific #88954).

### Flow Cytometry

FLS grown on soft dishes at 2 kPa (ExCellness) for 72 hours coated with fibronectin with various *stimuli* for 48 hours were collected by trypsination. RA FLS were incubated in 1X annexin V binding buffer with Annexin V detection kit (ab14155, Abcam) or permeabilized and fixed before 1h labeling with ki67 antibody. Data were acquired using a FACSCanto II cytometer and analyzed with the BD FACS Diva software 6.1.3 (BD Nova Biosciences, UK).

### Invasion Assay

RA FLS invasion abilities were assessed using BioCoat™ Growth Factor reduced Matrigel Invasion Chamber (Corning, Corning, NY, US). FLS were seeded in the upper chamber at 50,000 cells per insert in DMEM containing 1% glutamine, 1% PS and 0.1% BSA. DMEM containing 20% FBS, 1% glutamine, 1% PS and 0.1% BSA was added to the wells. The invasion chambers were then incubated for 48 hours with or without TNF, IL-17, and VP (which were added in the insert chamber and in the wells at the same concentrations). After incubation, noninvading cells were removed from the upper surface of the membrane, inserts were fixed in 4% PFA for 20 minutes at RT, and then stained with hematoxylin and eosin (H&E). Membranes of insert were imaged at x100 magnification and invading cells were counted. Results are represented by the mean of two inserts for each condition, and the mean of three images at x100 counting per insert.

### Organoids Culture and Processing

Synovial organoids assembly protocol was adapted from previous publication (31). Briefly, cells from OA or RA patients were used and mixed with Matrigel (Corning) at 4.10^6^ cells/ml of Matrigel, 22 µl droplets (representing approximatively 90,000 cells) were added to 96-well U-shaped very low attachment surface plates (Corning). Gelation was then allowed for 45 minutes at 37°C in a CO_2_ incubator. Then specific medium was added: DMEM supplemented with 10% FBS, 1% glutamine1% nonessential amino acids, 1% penicillin-streptomycin (PS), 0.1mM ascorbic acid, and ITS solution. Organoids were maintained for 21 days in culture media. VP with or without TNF and IL-17 were used at the same concentration as for cell culture. At day 21, organoids were fixed with glyoxal/ethanol solution for 1 hour at RT (PFA fixation being deleterious). Organoids were then embedded in paraffin, cut at 6µm and stained with H&E. Other organoids were embedded in a gelatin-sucrose solution and frozen in an isopentane bath at -50°C for 2 minutes before storage at -80°C. Thick cryosections (30µm) were then used for immunofluorescence labeling.

### Animals

#### General Information for Animal Experiments

Animals were tattooed and randomized into groups of equal weight. Experiments were performed after at least two weeks of acclimatization. All animal experiments were conducted by at least two independent experimenters, one of whom was blinded to the group allocation. There were no inclusion or exclusion criteria during animal experiments. No data exclusion was performed except if samples were impossible to use due to low quality (for example low RNA quality due to degradation). All animal studies were performed in accordance with the European Community legislation and approved by the Ethical Committee for Animal Experiments of Saint-Etienne University, agreement number: 2019032816186046 for rat AIA model and 2019032010448893 for DSS mice.

#### Animal Care

All animals were housed at 2 or 3 per cage in the PLEXAN facility with 12/12h light/dark cycles and *ad libitum* water and food access. In accordance with these guidelines, regulations, and 3Rs principles, specific enrichments were used to improve animal wellfare. Anticipated endpoints were predefined and never reached. No specific pain medication was used since it interferes with inflammatory response.

#### AIA Rats for mRNA Kinetics

For rat mRNA kinetics analysis, samples from our previous work were used with the same protocol (32), rats were killed at different time points (n=5 for each time point) after arthritis induction (at day 6, 8, 10, 12, 17, and 24) with five control animals for each time point.

#### Adjuvant-Induced Arthritis Rat

The arthritis (rat AIA n=21) was induced by 1.5 mg of *Mycobacterium butyricum* (Difco Laboratories, Detroit, MI, US) injection in 6-weeks old Lewis female rats (Charles River Laboratories, L’arbresle, France) as previously described (32). Control (non-AIA n=7) rats received vehicle injection without *Mycobacterium butyricum*. In the preventive group, daily intraperitoneal (IP) injections of VP (Tocris biosciences, Bristol, UK) at 20 mg/kg/days started at day 6 (before arthritis onset); whereas in the curative VP treatment group, daily IP injections of VP at 40 mg/kg/days started at day 12 (after arthritis onset). For all groups, IP injections started at day 6 (with or without VP) and were performed with 600 µL of vehicle containing 10% DMSO. All rats were followed as previously described (31). Rats were sacrificed at day 17, ankle and spleen were stored at -80°C for further analysis. At necropsy, the right ankle was frozen and stored for mRNA analysis. A synovial biopsy was performed on the left ankle, then the fragment of synovial membrane was fixed in 4% PFA for 20 minutes and stored in 10% ethanol solution at 4°C before AFM measurements. After synovial fragment collection, left ankles were fixed with 4% PFA solution for 48 hours at 4°C. Microcomputed tomography analysis was conducted prior to decalcification in 0.5 M EDTA. Spleens were sectioned into two pieces, one for mRNA analysis and the second was fixed in 4% PFA. Spleens and decalcified ankles were next cryoprotected, then embedded and frozen at -80°C. Sections were performed using Microm HM 560 cryostat (Thermo Fisher Scientific).

#### Dextran Sulfate Sodium Induced Colitis Mice

Eight-weeks old female Balb/cByJ mice (Charles River Laboratories) were treated with an IP injection of VP at 40 mg/kg/day (100 µL, in 10% DMSO) or 10% DMSO one day prior to the beginning of DSS (MP Biomedicals) treatment. The latter was administered at 1.5% in drinking water, fresh solutions were prepared and changed every day for 11 days (n=14). Control mice received only drinking water and an injection of 10% DMSO (n=7). For all groups, daily IP injections were continued during DSS treatment until mice were sacrificed at day 11. Disease activity index scoring comprised several scales evaluating physiological and behavioral parameters including percentage of weight loss, stool consistency, evidences of intestinal leakage with the presence of blood in stool, animal general aspect and animal pain behavior. Scoring and weighing were performed each day. After death, intestinal tracts of mice were dissected and cut into pieces with each containing a Peyer’s patch. Two pieces were snap frozen for further mRNA analysis and two other pieces were placed in 4% PFA at 4°C for 48 hours, embedded and frozen in the same process than rat AIA ankles.

### RNA Extraction and RT-qPCR

For animal tissues (spleen, gut and ankle) and synovial organoids lysis was performed in TRI Reagent (Sigma). To obtain enough RNA, 3 synovial organoids were pooled for lysis. RNA was purified with RNeasy plus (Qiagen Inc.). For cell culture, RNA was extracted using Allprep RNA/protein kit (Qiagen Inc.). Quality and quantity of RNA were assessed by Experion RNA analysis (BioRad) and QuantIT RiboGreen RNA assay (Thermo Fisher Scientifc), respectively. Complementary DNA (cDNA) was synthesized using the iscript cDNA synthesis kit (Biorad). Quantitative RT polymerase chain reaction (PCR) was conducted on CFX96 RealTime System (BioRad) with LightCycler FastStart DNA Master plus SYBRgreen I (Roche Diagnostics, Basel, Switzerland). The results were normalized to the housekeeping gene expression hypoxanthine-guanine phosphoribosyltransferase (HPRT).

### Immunofluorescence

This technique was done on frozen sections (organoids, spleen and ankle of rats, and mice gut) or on RA FLS and HEK293 cells fixed with 4% PFA at RT for 20 minutes. For RORγT (Biorbyt, San Francisco, CA, US) labelling on spleen and gut, citrate antigen retrieval was performed. Before labelling, sections or cells were rehydrated, permeabilized in 0.3% Triton X-100, then blocked in 1% BSA 5% goat serum and 0.1% Triton solution for 60 minutes at RT, and probed with the primary antibody diluted in the blocking solution overnight at 4°C. The following primary antibodies were used: YAP (63.7 sc-101199, Santa Cruz Biotechnology; 1:100), YAP (D8H1X, Cell Signaling Technology; 1:100), Ki67 (NB-110-89717, Novus Biologicals, Abingdon, UK; 1:100), c-Jun (9165, Cell Signaling Technology, 1:200), and tenascin-C (ab215369, Abcam, 1:100). After washing, sections or cells were incubated with secondary antibodies, goat anti-rabbit rhodamine coupled antibody (31686, Thermo Fisher; 1:300) and/or goat anti-mouse 488 (A11034, Thermo Fisher) for 75 minutes at RT, all diluted at 1:400. Slides or cells were counterstained with DAPI alone (10 minutes at 37°C) or coupled with phalloidin (R415, Thermo Fisher; or ab176753, Abcam) for 1 hour at 37°C. Isotypic controls were always performed using rabbit IgG isotype control (31235, Thermo Fisher) and mouse IgG isotype control (31903, Thermo Fisher), diluted at the same concentration as the primary antibody.

### Image Acquisition, Analysis, and Quantification

Images were acquired using a confocal laser microscope (LSM) 800 airyscan (Zeiss, Oberkochen, Germany) equipped with Zen software. This microscope was used in epifluorescence, classic confocal or airyscan confocal mode depending on the needs. Comparison between groups for image analysis and quantification were performed using the exact same settings from antibodies labelling to image processing.

#### Immunofluorescence Images Quantification

Ki-67 and YAP labelling on RA FLS were evaluated by automatically defining nuclear region of interest (ROI) area based on DAPI staining, ROI nuclear mask was then added on YAP and Ki67 images. Positive cells for YAP or Ki67 labelling in the nucleus were then counted and divided by the total number of nuclei (DAPI). This quantification was performed on large acquisition tiles at x100 magnification, representing approximately 50 to 200 cells per well. C-Jun labelling on frozen sections of organoid was quantified by measuring mean intensity in DAPI ROI in the synovial lining layer divided by the same measurement in the stroma.

#### Organoid Synovial Hyperplasia

This quantification was performed on H&E paraffin sections. Each organoid assessment was the result of two organoid slices. Images were binarized and a synovial lining layer area was automatically selected. Organoid perimeters were also measured. Hyperplasia criteria was the result of synovial lining layer area divided by synovial organoid perimeter.

#### Peyer’s Patches Area

This quantification was performed on H&E frozen intestinal sections. Area of Peyer’s patches was evaluated by manual ROI drawing based on histology. For each mouse, result was the mean of two distinct Peyer’s patches (one from the beginning and one from the end of the intestinal tract).

### Microcomputed Tomography

Rat AIA ankles were scanned *ex vivo* with vivaCT40 (Scanco, Brüttisellen, Switzerland) at 55 kVp (peak kilovoltage) and reconstructed under a resolution of 12.5 µm. Quantification and 3-D imaging were performed after reconstruction. Reconstruction was performed under 0.5/2/307 (Gauss sigma/Gauss support/lower threshold).

### AFM Measurement

AFM measurements were performed using standard force mode on a Veeco Multimode AFM equipped with Nanoscope IIIa controller and Picoforce extension. Briefly, we glued a 50-µm polystyrene microsphere (Alpha Nanotech, Morrisville, NC, US) on a 0.1 N/m rigidity cantilever (Bruker MLCT-Bio, E triangular) and this cantilever was used for all measurements. Rat synovial samples were dissected under binocular microscope from the same area. A nano-indentation was performed in force mode by recording two force curves on three independent areas twice for each sample. The acquired force curves were exported in ASCII format with nanoscope V614r1 software, and then the data were processed using a proper Matlab^®^ code. The code includes the fitting of the contact area from the force curves with a Hertz contact model in order to extract Young’s elastic modulus (rigidity in kPa).

### Statistical Analysis

Data were represented as single values, with mean and standard deviation or median and interquartile range, accordingly to normality. Some parameters were expressed as percentage of the mean of control values. Two comparisons were done with Mann-Whitney test or unpaired Student t-test. Multiple comparisons were performed by ANOVA or Kruskal-Wallis test, *post hoc* comparisons were corrected with the false discovery rate method of Benjamini and Hochberg (q-values). Results were considered significantly different when p<0.05 or q<0.05. All statistical analyses were performed on GraphPad Prism 8.2.0 software.or.

## Results

### High YAP/TAZ Transcriptional Activity During Arthritis

**Figure 1:**
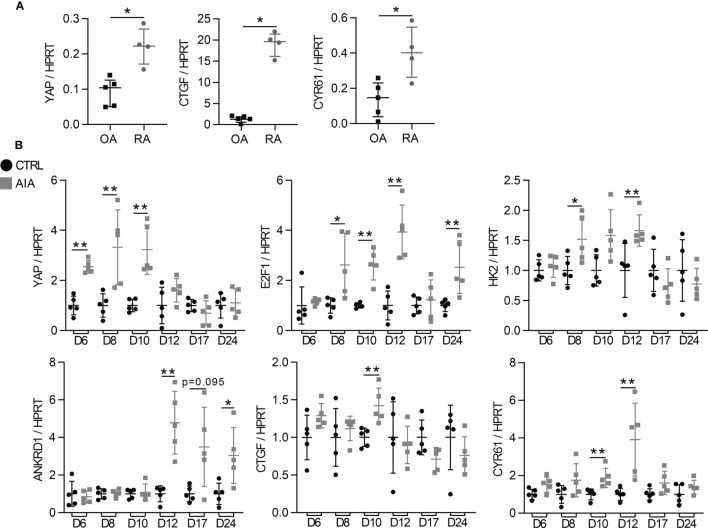
High YAP/TAZ transcriptional activity during arthritis **(A)** RT-qPCR quantification of YAP, CTGF and CYR61 expression normalized to HPRT expression in synovial organoids at 21 days of culture. Organoids (n=12 to 15) were formed with FLS from one rheumatoid arthritis (RA) and one osteoarthritis (OA) patient. Each point represents three organoids that were mixed for RNA extraction. **(B)** RT-qPCR results of mRNA from the ankle of adjuvant induced arthritis (AIA) and control (CT) rats. Results were normalized to HPRT expression and expressed as fold change vs. CT at each day. Rats were sacrificed at different times (n=5) with an arthritis onset observed at day 10. Data were presented as individual values with median and interquartile range. Mann-Whitney tests were used. D, day post induction; YAP, yes-associated protein; HPRT, hypoxanthine phosphoribosyltransferase; CTGF, connective tissue growth factor; CYR61, cysteine-rich angiogenic inducer 61; E2F1, E2F transcription factor1; HK2, hexokinase2; ANKRD1, ankyrin repeat domain-containing protein1; *p < 0.05; **p < 0.01.

To investigate YAP/TAZ activity during arthritis, FLS from RA patients were used and compared to FLS from osteoarthritis (OA) patients as controls. As YAP/TAZ transcriptional activity was reported activated by substrate stiffness (such as classic culture dishes), YAP/TAZ activity was compared between RA and OA FLS using a synovial organoid model. This model recapitulated features of the synovial membrane *in vivo* and was closer to physiological conditions than 2D cell culture. Compared to OA organoids, RA synovial organoids displayed higher expression of YAP and its target genes: connective tissue growth factor (CTGF) and cysteine-rich angiogenic inducer 61 (CYR61; **Figure 1A**). The high *in vitro* YAP/TAZ transcriptional activity was then confirmed *in vivo* using the adjuvant-induced arthritis (AIA) rat model (32). In this model, the mRNA level of YAP increased in the ankle of AIA rats on days 6, 8 and 10 after induction (before arthritis onset), followed by an increased expression of its target genes, including E2F transcription factor 1 (E2F1), hexokinase 2 (HK2), ankyrin repeat domain-containing protein 1 (Ankrd1), CTGF and CYR61 on days 10 and 12 after induction (at the arthritis onset; **Figure 1B**).

### Increased YAP/TAZ Transcriptional Activity in RA FLS by Inflammation and Mechanical Priming

**Figure 2:**
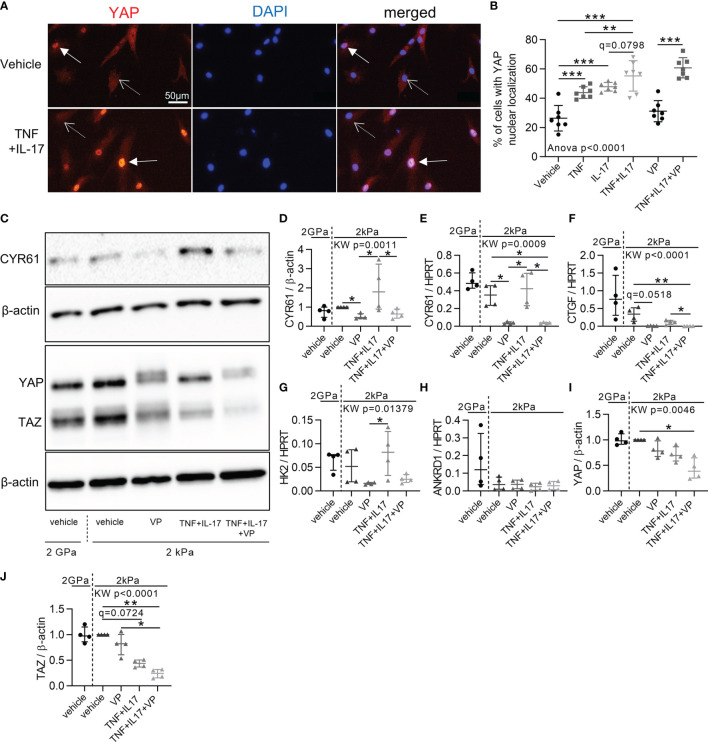
Increased YAP/TAZ transcriptional activity in RA FLS by inflammation. RA FLS (n=4 patients) were cultured on soft (2 kPa) or classic stiff (2 GPa) substrates as indicated with VP, TNF, IL-17 alone or in combination for 48 hours. **(A)** Epifluorescence representative images of YAP (immunofluorescence, orange), DAPI (nucleus, blue), and merged images of RA FLS in 2kPa conditions. Arrows: positive YAP nuclear localization; dotted arrows: negative YAP nuclear localization. **(B)** Corresponding quantification for FLS from one RA patient (n=7). Experiments were repeated for FLS for three others RA patients showing the same pattern. **(C)** Representative western blot results of total YAP-TAZ, CYR61, and β-actin with their quantifications normalized to the 2 kPa group for each patient and to β-actin **(D, I, J)**. **(E–H)** RT-qPCR quantifications of CYR61, CTGF, HK2 and Ankrd1 expression normalized to HPRT expression. Data were presented as individual values with mean ± SD **(B)** or median and interquartile range **(D–J)**. ANOVA or Kruskal Wallis (KW) test with FDR corrected (q-value) for multiple comparisons *post hoc* tests between soft conditions (2kPa): *q < 0.05; **q < 0.01; ***q < 0.001. Verteporfin (VP), 10 µM; tumor necrosis factor (TNF), 10 ng/ml; Interleukin-17 (IL-17), 50 ng/ml; TAZ, transcriptional co-activator with PDZ-binding motif.

Then, to explore the mechanisms involved in YAP/TAZ transcriptional activity in RA FLS, we first focused on inflammation effect. TNF and IL-17, two pro-inflammatory cytokines strongly involved in RA, were used to mimic the *in vivo* inflammatory environment of RA (33, 34). To avoid substrate stiffness-induced YAP activity, RA FLS were grown on soft substrate (2kPa). Nevertheless, classic support stiffness (2GPa) was kept as reference. TNF or IL-17 increased YAP nuclear localization at 48 hours in RA FLS with a synergistic effect (**Figures 2A, B**). Consistently, CYR61 protein level trended to increase upon cytokines treatment in RA FLS (**Figures 2C, D**), whereas the mRNA expression of YAP target genes was not significantly enhanced at 48 hours (**Figures 2E, H**). After TNF and IL-17 stimulation, YAP total protein was unchanged in RA FLS, while total TAZ protein trended to decrease (**Figures 2I, J**).

Despite absence of inflammation or substrate stiffness, YAP remained nuclear in some RA FLS (**Figures 2A, B**). Consequently, mechanical priming of FLS by stiff environment associated with a possible persistent YAP activation was explored, since this concept was already reported in mesenchymal stem cells (35). For this purpose, RA FLS were grown on stiff substrate (classic culture substrate 2 GPa) up to passage (P) 3, P4, or P5, then switched to soft substrate for 72 hours after each of these passages (P4, P5 or P6, respectively; **Supplementary Figure 1A**). YAP nuclear localization was higher at P5 than P4 (**Supplementary Figures 1B, C**) with higher ANKRD1 expression at P6 compared to P5 (**Supplementary Figure 1D**). Thus, time spent by RA FLS on stiff substrate enhanced YAP activity when assessed on a soft substrate suggesting that mechanical stiffening could promote YAP autonomous activity in FLS. Notably, inflammation and mechanical *stimuli* activated different YAP target genes when applied separately but had synergistic effects when applied together by increasing YAP target genes expression (**Supplementary Figures 1D, E**). Taken together, these results indicated that inflammation and mechanical stiffening were inducers of YAP/TAZ transcriptional activity in RA FLS.

### Reduction of RA FLS Aggressive Phenotype by YAP/TAZ Inhibition

**Figure 3:**
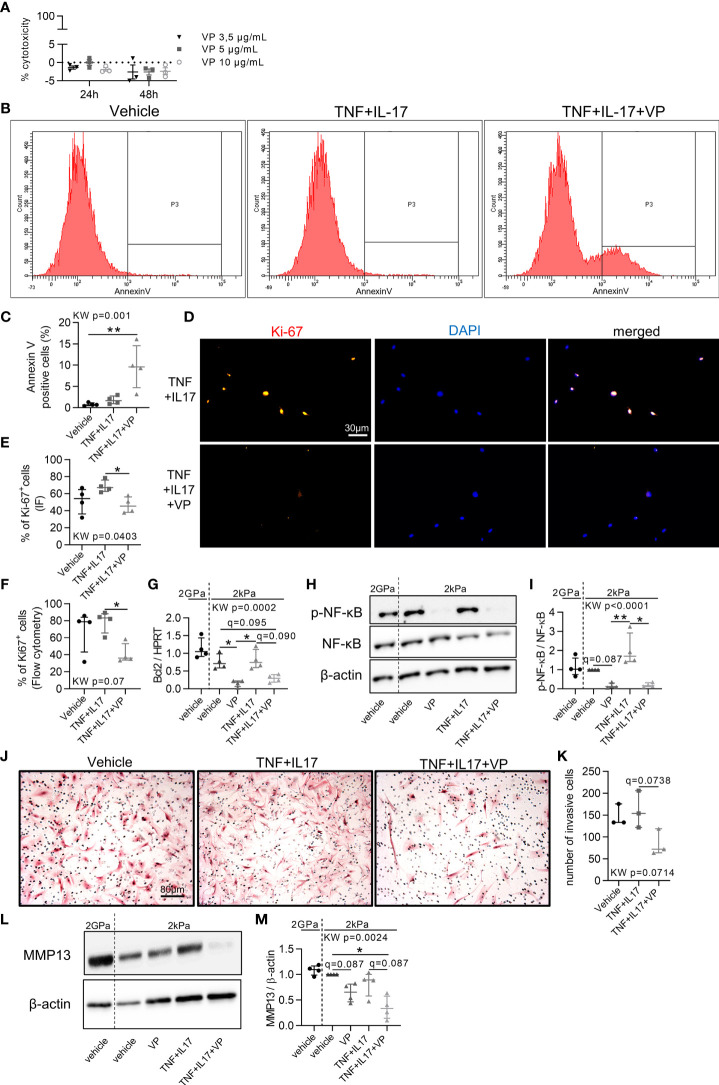
Blocking the aggressive phenotype of RA FLS by YAP/TAZ inhibition. RA FLS (n=4 patients, except when specified) were cultured on soft (2 kPa) or classic stiff (2 GPa) substrate and treated with VP, TNF, IL-17 for 48 hours. **(A)** Lactate dehydrogenase cytotoxicity assay for RA FLS (n=1 patient) in triplicate. Positive control was set at 100%. **(B, C)** Flow cytometry representative histograms **(B)** showing the number of annexin V negative (left) and positive cells (right) with corresponding quantification **(C)**. **(D, E)** Representative epifluorescence images **(D)** of Ki-67 (orange), DAPI (blue), and merged with corresponding quantification **(E)** and flow cytometry quantification **(F)**. **(G)** RT-qPCR quantification of Bcl2 expression normalized by HPRT expression. **(H, L)** Representative western blot results of phospho(p)-NF-κB p65 (serine536: active form), total NF-κB p65, β-actin **(H)**; and MMP-13 **(L)** with their quantification normalized by the 2kPa group for each patient and β-actin **(I, M)**. **(J, K)** Representative images of invasion transwell assay stained with hematoxylin and eosin (H&E) at x10 magnification **(J)** with quantification **(K)** performed in duplicate for RA FLS (n=3 patients). Data were presented as individual values with median and interquartile range. For statistical analysis, please see Figure legend 2. VP, 10 µM (except other mention); TNF, 10 ng/ml; IL-17, 50 ng/ml; p, phospho; NF-κB, nuclear factor-κB; MMP, matrix metalloproteinase.

Since YAP/TAZ transcriptional activity was high in RA FLS, its inhibition with VP was explored to restore a normal phenotype in control or inflammatory conditions. First, VP treatment had no effect on YAP nuclear translocation (**Figure 2B**), but reduced YAP target genes levels (**Figures 2C** **–H**) blocking YAP transcriptional activity, as did a YAP siRNA on CYR61 expression (**Supplementary Figure 2A**). VP did not affect cell mortality (**Figure 3A**). However, apoptotic annexin V positive cells were increased by the combination of VP with TNF and IL-17 (**Figures 3B, C**). Consistently, the number of Ki-67 positive (proliferating) RA FLS was decreased by VP treatment in the presence of TNF and IL-17 assessed by two independent methods: immunofluorescence (**Figures 3D, E**) or flow cytometry (**Figure 3F**). At molecular level, changes in the proliferation/apoptosis balance were associated with decrease of Bcl2 expression in VP treated groups with or without inflammatory conditions (**Figure 3G**) and in YAP siRNA RA FLS with inflammatory conditions (**Supplementary Figure 2B**). Furthermore, VP strongly reduced the phospho(p)-NF-κB (phosphorylation on serine 536, active form involved in survival response)/total NF-κB ratio with or without TNF and IL-17 treatment (**Figures 3H, I**). YAP siRNA reduced p-NF-κB/β-actin, but not the p-NF-κB/NF-κB ratio (**Supplementary Figures 2C–F**). Finally, VP trended to decrease the invasion ability of RA FLS even in pro-inflammatory conditions (**Figures 3J, K**) and MMP-13 protein level when TNF and IL-17 were co-administered with VP in RA FLS (**Figures 3L, M**). So, blocking YAP transcriptional activity strongly reduced aggressive RA FLS phenotype.

### Prevention and Reversal of Synovial Hyperplasia in a Synovial Organoid Model by YAP/TAZ Inhibition

**Figure 4:**
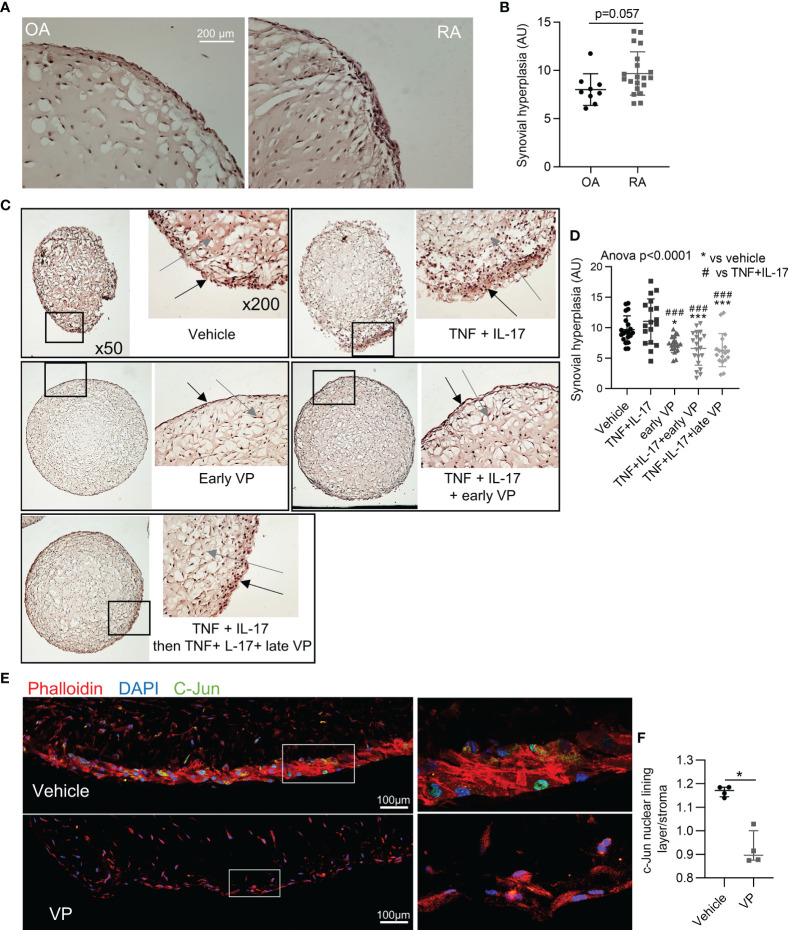
Prevention and reversal of synovial hyperplasia in a synovial organoid model by YAP/TAZ inhibition. **(A, B)** Representative images at x200 magnification after H&E staining of synovial organoids **(A)** with quantification **(B)** of synovial hyperplasia in organoids (n=9 for OA and 20 for RA) from RA (4 patients) and OA (3 patients) FLS. **(C)** Representative images at x50 and x200 magnification after H&E staining sections of synovial organoids with RA FLS from the same patient; black arrows: synovial lining layer, grey arrows: synovial stroma. **(D)** Corresponding quantification of synovial hyperplasia in organoids (n=15 to 20 per group) from RA FLS (n=4 patients). * comparisons vs. vehicle, # comparisons vs. TNF+IL-17. **(E)** Representative confocal tiles images at x630 for c-Jun (green), phalloidin (actin, red), and DAPI (nucleus, blue) on RA organoid with corresponding zoom (right). **(F)** Ratio of nuclear c-Jun for cells in lining layer and nuclear c-Jun for cells in the stroma (organoids n=4 from FLS of one RA patient). Data were presented as individual values with mean ± SD **(B, D)** or median with interquartile range **(F)**. T-test **(B)**, ANOVA test with FDR *post hoc* test corrected (q-value) for multiple comparisons **(D)** or Mann-Whitney **(F)**. *q or p < 0.05; ^###^q < 0.001). VP, 10 µM; TNF, 10 ng/ml; IL-17, 50 ng/ml; AU, arbitrary units.

YAP has been described to promote synovial hyperplasia following cartilage injury in mouse model (36). Here, YAP inhibition was explored to prevent and to reverse synovial hyperplasia in RA. To mimic such synovial hyperplasia, we used the synovial organoid model. After 21 days of culture, RA FLS were able to form a thick lining layer, which trended to be thicker than those formed by osteoarthritic (OA) FLS independently of any inflammatory *stimuli* (**Figures 4A, B**). Since RA FLS organoids were already hyperplasic without any *stimuli*, TNF and IL-17 did not increase further synovial hyperplasia (**Figures 4C, D**). VP treatment from the start of organoid formation prevented hyperplasia in basal and inflammatory conditions (**Figures 4C, D**). In addition, VP treatment starting at day 14 (when synovial hyperplasia was already observed) also inhibited hyperplasia formation, suggesting its potential effect to reverse synovial hyperplasia, consistent with a curative approach (**Figures 4C, D**). To explore signaling pathways involved in hyperplasia formation, c-Jun subcellular localization was investigated. Nuclear localization of c-Jun in FLS was higher at the “lining layer” site compared to the “stroma” site and VP treatment suppressed this difference suggesting that YAP/TAZ have a role in the regulation of c-Jun in this context (**Figures 4E, F**).

### Prevention and Reduction of Arthritis Severity in Rat AIA Model by YAP/TAZ Inhibition

**Figure 5:**
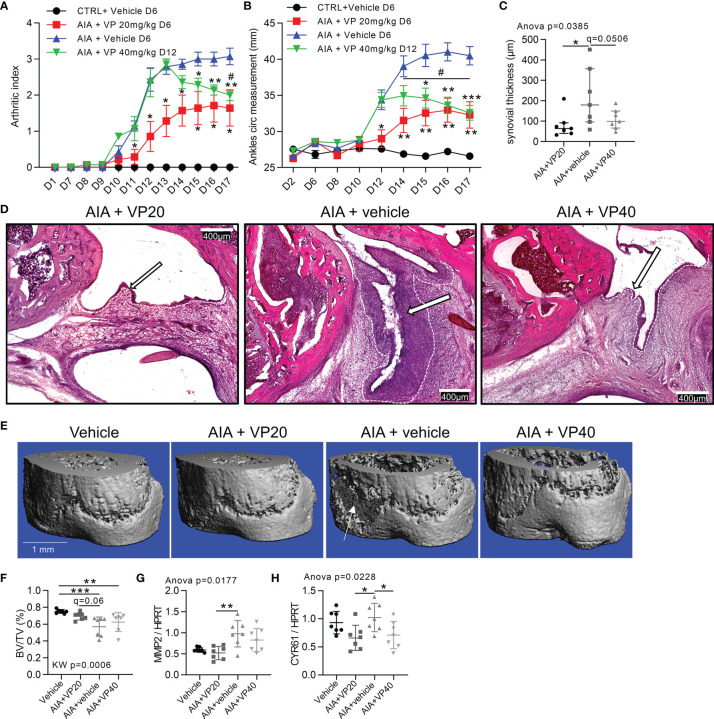
Prevention and reduction of arthritis severity in rat AIA model by YAP inhibition. Day 0 corresponded to AIA induction. Control or arthritic (AIA) rats were IP injected with vehicle containing 10% DMSO, treated AIA rats received 20 µg/kg/day of VP from day 6 (VP20) as a preventive approach or 40 µg/kg/day VP from day 12 (VP40) as a curative approach. **(A, B)** Arthritic index and ankles circumference measurements. Two-way ANOVA with FDR *post hoc* test corrected for multiple comparison (*), and paired t-test between days 14 and 17 (#) for VP40 group. **(C, D)** Quantification of synovial hyperplasia **(C)** with representative tiles images at x100 magnification **(D)**, H&E staining; arrows: synovial lining layer; dotted lines: limit between synovial lining layer and synovial stroma. **(E, F)** Representative 3-D reconstruction of micro-computed tomography scan for distal tibia epiphysis **(E)** with corresponding quantification **(F)** of BV/TV (bone volume/tissue volume); arrow: bone erosion. G-H: RT-qPCR quantification of MMP2 **(G)** and CYR61 **(H)** expression normalized to HPRT expression. Data were presented as individual values with mean ± SEM **(A, B)** or mean ± SD **(C, F–H)**. Kruskal Wallis **(F)** or ANOVA test with FDR *post hoc* test corrected (q-value) for multiple comparisons. * or # q < 0.05; **q < 0.01; ***q < 0.001.

To assess the involvement of YAP/TAZ in arthritis onset and severity, VP was delivered in AIA rats to explore preventive and curative approaches. Preventive VP injection from days 6 to 16 (starting before arthritis onset) induced a delay in arthritis onset, with a strong reduction in arthritis severity and ankles circumference compared to AIA vehicle rats (**Figures 5A, B**). Curative VP injection from days 12 to 16 (starting after arthritis onset) completely blocked arthritis progression and reduced arthritis severity with a decrease of both ankles circumference and arthritic index (**Figures 5A, B**). Thus, VP curative approach allowed regression of arthritis clinical signs. The reduction of arthritis severity was directly linked with a reduction of synovial hyperplasia observed in both VP groups at day 17 (**Figures 5C, D**), confirming our previous *in vitro* data and reinforcing the key role of YAP/TAZ activity for hyperplasia formation and maintenance. Bone volume per tissue volume (BV/TV) was decreased in AIA rats, corresponding to bone erosion, and linked with FLS invasion (**Figures 5E, F**). Strikingly, this bone loss was avoided in the preventive VP treatment, but not in the curative approach. In the preventive approach, the bone protection was associated with a reduction of MMP2 expression compared to the AIA group (**Figure 5G**), thus corroborating the reduced invasion ability of FLS under VP treatment. CYR61 expression was reduced in both VP injection protocol (**Figure 5H**), confirming VP effect on YAP/TAZ transcriptional activity.

### Decreased Inflammatory Markers *In Vivo* and *In Vitro* Induced by YAP/TAZ Inhibition

Then, anti-inflammatory effect of systemic YAP/TAZ inhibition with both VP approaches was evaluated. RORγT labelling was detected in the marginal zone of spleen from AIA rats and strongly reduced in both VP groups (**Supplementary Figure 3A**). In the spleen, IL17 mRNA was reduced in the preventive VP group, with a similar trend in the curative VP group (**Supplementary Figure 3B**). In the ankle, IL17 mRNA was reduced only in the preventive VP group (**Supplementary Figure 3C**). No differences in IL10 expression (**Supplementary Figure 3D**) were observed in the spleen, suggesting that VP had no effect on Tregs. Unexpectedly, TNF expression was lower in spleen of VP treated animals, with no change in the ankle (**Supplementary Figures 3E, F**), whereas no change in IL6 expression was observed (**Supplementary Figures 3G, H**). Consequently, the effect of VP in reducing IL-6 or TNF expression by RA FLS was then investigated *in vitro*. TNF and IL-17 treatment increased both IL6 and TNF expressions in RA FLS (**Supplementary Figures 3I, J**). However, VP trended to inhibit both TNF and IL6 expression in RA FLS treated with or without TNF and IL-17 (**Supplementary Figure 3I, J**). To conclude, inhibiting YAP/TAZ transcriptional activity with VP in arthritis has immunomodulatory systemic effect by acting on both immune and non-immune cell types.

### Regulation of Mechanical Changes in the Synovial Membrane During Arthritis Through YAP/TAZ Transcriptional Activity

**Figure 6:**
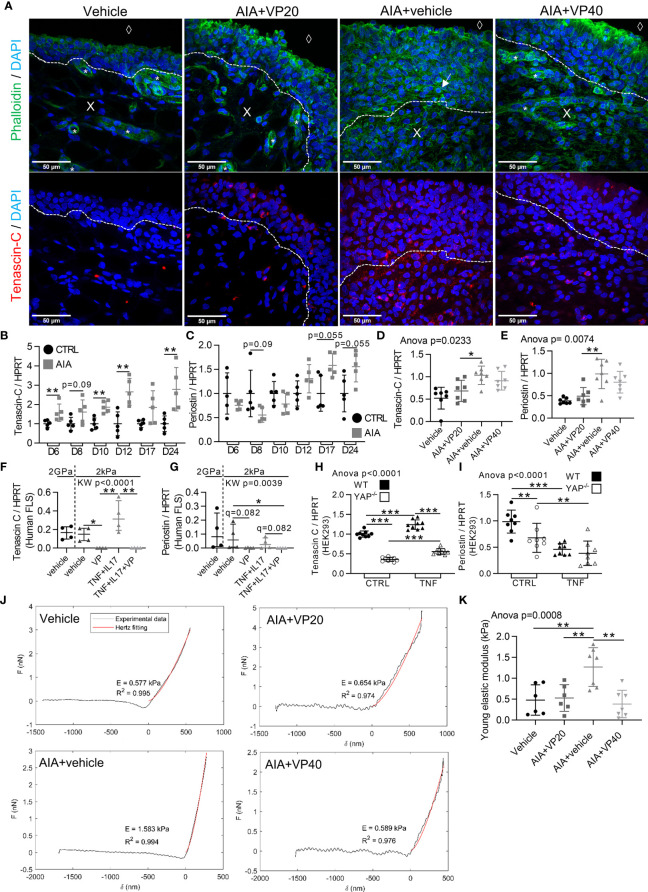
Increased stiffness of synovial tissue during arthritis under the control of YAP/TAZ. Samples used for *in vivo* experiments were described in **Figures 1** and **4**. RA FLS (n=4 patients) were used as described in **Figure 2**. HEK293 results are representative of three independent experiments (n=9 per group). **(A)** Representative confocal tiled images of tenascin-C (red), counterstained with DAPI (blue) and phalloidin (green) in synovial membrane of rat AIA model; dotted lines: limit between synovial lining layer and synovial stroma; X: stromal compartment; diamond: synovial fluid compartment; star: blood vessels; arrow: actin stress fiber. **(B–I)** RT-qPCR results for tenascin-C and periostin in AIA and control rats ankle with different kinetics **(B, C)** VP AIA treated rats ankle **(D, E)**; RA FLS **(F, G)** or HEK293 WT and YAP^-/-^ cells **(H, I)**. **(J)** Representative punctual nanoindentation curves for rat synovial membrane using AFM. Increased slope corresponded, when fitted with Hertz model (red), to an increased Young’s modulus (indicated as **E**) **(K)** Corresponding quantification of Young’s modulus (kPa). Data were presented as individual values with mean ± SD **(D, E, H, I, K)** or median and interquartile range **(B, C, F, G)** with Kruskal Wallis or ANOVA test with FDR *post hoc* test corrected (q-value) for multiple comparisons *q < 0.05; **q < 0.01; ***q < 0.001. TNF, 10ng/ml.

Since YAP/TAZ activity was crucial during arthritis and YAP/TAZ activity could be regulated by mechanical changes in tissues, synovial mechanical properties during arthritis were investigated. In synovial membrane of AIA rats (**Figure 6A**), SF formations were observed. Strikingly SF formation was absent with VP treatments, suggesting that YAP could contribute to increase FLS tension. Consequently, the involvement of YAP in controlling the expression of ECM stiffness-related components was explored by focusing on tenascin-C and periostin (29). A strong tenascin-C (**Figure 6A**) and periostin (not shown) labelling was observed in the sublining area of the synovial membrane in non-treated AIA rats, but not in control and VP treated animals. Furthermore, tenascin-C expression was increased before and after arthritis onset, whereas periostin expression trended to increase only after arthritis onset in AIA rats ankles (**Figures 6B, C**). The decrease in tenascin-C and periostin was confirmed at the mRNA level in preventive VP approach (**Figures 6D, E**). The same pattern was observed *in vitro* for VP treated RA FLS (**Figures 6F, G**). To further confirmed that these two genes are specifically regulated by YAP transcriptional activity, YAP deficient (YAP^-/-^) HEK293 cells obtained with CRISPR-cas9 technique were used. Both tenascin-C and periostin expression were strongly reduced in YAP^-/-^ HEK293 (**Figures 6H, I**). Additionally, using chromatin immunoprecipitation followed by next-generation sequencing (ChIP-seq) data from another report (37), three YAP/TAZ/TEAD4 peaks were found at active enhancer sites of tenascin-C gene (37). Together these results indicated that tenascin-C and periostin were YAP direct target genes. Next, to explore whether these changes in cytoskeletal and ECM composition could influence synovial stiffness, the Young’s modulus, also known as the sample stiffness, was assessed in the rat AIA model. Synovial stiffening during arthritis was strongly increased in AIA rats. Furthermore, no synovial membrane stiffening was observed in both preventive and curative VP approaches (**Figures 6J, K**). These results showed that YAP transcriptional activity mediated pro-fibrotic responses and synovial stiffening in arthritis.

### Prevention of Colitis in a Mouse Model by YAP/TAZ Inhibition Through Similar Mechanisms Observed in Arthritis

To extend our results in another inflammatory environment, we focused on colitis. Indeed, YAP/TAZ activity has already been described in IBD. VP decreased disease severity in a mouse colitis model (11). In this context, we focused on another colitis model, the dextran sulfate sodium (DSS) induced colitis mouse model and confirmed VP efficacy with a reduction of colitis severity (**Supplementary Figure 4A**). As previously shown for FLS or synovial tissue, VP also trended to downregulate CYR61 expression at the gut level (**Supplementary Figure 4B**). Histologically, intestinal *villi* appeared less damaged in the VP group, and the area of Peyer’s patches, a witness of intestinal inflammation was reduced by VP treatment (**Supplementary Figures 4C–E**). VP treatment decreased gut TNF expression with no change for IL6 (**Supplementary Figures 4F, G**), thus reflecting the immunomodulatory effect of VP described in arthritis. However, no difference in IL17 expression during colitis was observed in the gut (**Supplementary Figure 4H**). MMP2 expression was also reduced under VP treatment with a similar trend for MMP13 (**Supplementary Figures 4I, J**). Finally, tenascin-C expression also trended to increase in DSS treated mice, whereas this was not the case in VP treated mice (**Supplementary Figure 4K**), suggesting that tissue stiffening could also occurred through YAP during colitis.

## Discussion

YAP/TAZ have been shown to be active in an increasing number of inflammatory related diseases especially in IBD (10–13, 25), but also in cancers where inflammation is also involved (2). Thus, we demonstrated that pro-inflammatory cytokines enhanced YAP/TAZ nuclear translocation and transcriptional activity, and this confirmed the relevance of YAP/TAZ targeting for therapeutic purpose in our arthritis and colitis models, but also possibly in other inflammatory diseases. Our study provided new and deeper *in vitro* and *in vivo* evidence on YAP/TAZ involvement during arthritis. Here, we focused mainly on YAP/TAZ-TEAD transcriptional activity which was targeted by VP (4, 24). This high transcriptional activity impacted the RA FLS phenotype and could be directly connected with YAP target genes function including Bcl-2 and NF-κB modulation for apoptosis/proliferation regulation or tenascin-C for tissue stiffening. CYR61 and CTGF were highly expressed in our arthritic models, whereas YAP transcriptional activity inhibition strongly reduced their expression. Both CYR61 and CTGF were already extensively considered in arthritis (without previously reported link with YAP activity) and involved in RA pathophysiology and critical for FLS aggressive phenotype (38–42). Furthermore, inhibiting YAP-TEAD interaction also blocked some of the c-Jun/AP-1 transcriptional activity (37, 43). The inhibition of c-Jun nuclear localization by VP treatment in the lining layer of our RA organoid model reinforced the AP-1 modulation by YAP.

YAP was not only a responder to inflammation, since its blocking decreased pro-inflammatory response, as highlighted *in vitro* and *in vivo*. Several studies have also demonstrated that YAP transcriptional activity could increase the expression of pro-inflammatory cytokines such as IL-6 (10), but also key inflammatory mediators for immune cells recruitment such as CCL2 and IL-8 (44, 45). In our model, since VP strongly changed resident cells phenotype *in vivo*, including the expression of pro-inflammatory cytokines, it could possibly prevent the inflammatory process by inhibiting immune cell recruitment which could be mediated by FLS activity (17, 18). Consequently, healing of tissue resident cells could prevent inflammation runaway, thus being a possible mechanism in our *in vivo* models for the anti-inflammatory effect of VP. Thus, to better understand the specific role of YAP in different cell types (FLS and immune cells) for arthritis onset and severity, lineage specific KO of YAP could be performed in the future.

YAP/TAZ are also well-known to be activated by mechanotransduction events, like mechanical stress, actin dynamics process (26, 27, 45), and ECM stiffness (27). To avoid, mechanic YAP/TAZ activation, our *in vitro* 2D investigations were performed on soft substrate or in 3D with synovial organoid model. These approaches allowed us to described modulations of YAP independently of the non-physiological stiffness of classic culture dishes (2GPa). Despite that mechanical stiffness could be a methodological bias to study YAP/TAZ *in vitro*, mechanical changes are often found in pathological conditions *in vivo*, especially during inflammatory processes. In chronic inflammatory processes, the mechanical change in tissue allows YAP/TAZ activation (29). Here, we found that YAP/TAZ were indeed responders to mechanotransduction events, but they also strongly modulated mechanical properties of synovial tissue. VP treatment prevented the formation of actin stress fibers in both synovial organoid model and synovial membrane of AIA rats, suggesting that YAP is essential to respond to the tensive signal induced by inflammation. We also unraveled that YAP drove tenascin-C expression probably contributing to the stiffening process observed in synovial membrane of AIA rats. Strikingly, in addition to the role of tenascin-C in ECM stiffness, it has also been reported to be highly expressed in RA patients and to modulate chronic inflammation in models of RA (46), reinforcing again the concept to target YAP during RA. It appears clearly that mechanical changes are closely related to inflammatory processes. Thus, our study suggested that YAP could be the missing link between those two processes by converting inflammatory *stimuli* into cellular and tissue mechanical remodeling.

Overall, we reported a new potential signaling loop, where YAP/TAZ drove cellular tension and ECM stiffness through a strong change in ECM composition in response to inflammation, thus creating a stiffer micro-environment. It could in turn maintains YAP activity over time even when inflammation has stopped, since RA FLS could be mechanically primed. Such a vicious loop could explain the phenotype of RA FLS, which kept aggressive features even if they were in a normal environment (eg. non-inflammatory), and the synovial hyperplasia persistence in RA patients treated with anti-inflammatory drugs (47). Since targeting YAP/TAZ modulated the mechanical environment of cells, their modulation could effectively restore the mechanical properties of tissues. Interestingly, T lymphocytes responses could be potentiated by stiffness *in vitro* (48), meaning that restoring rigidity of the tissue with VP could decrease inflammation through mechanotransduction in T lymphocytes such as Th17. Additionally, this “self-maintained” YAP activity due to inflammation was probably also present in other inflammatory conditions such as IBD in which intestinal fibrosis has been described (49).

To conclude, our work shed light on YAP/TAZ role in arthritis, which could be also relevant for a broad range of inflammatory related events including cancer and inflammatory disorders research and therapy.

## Data Availability Statement

The datasets generated and analyzed during the current study are available from the corresponding author on reasonable request.

## Ethics Statement

The animal study was reviewed and approved by The Ethical Committee for Animal Experiments of Saint-Etienne University, agreement number: 2019032816186046 for rat AIA model and 2019032010448893 for DSS mice.

## Author Contributions

RC and HM designed the study. RC performed most of the experiments and contributed to all of them. EA performed WB techniques and contributed to mRNA and protein extraction. GC contributed to AIA rat mRNA kinetics and RA cell harvesting. MC contributed to the organoid model experiments. CP and LN contributed to AFM experiments. EM contributed to DSS mice experiments. MT contributed to RT-qPCR experiments. HM and AV-B contributed to rat AIA model handling. M-TL designed the mRNA primers. RC, EA, MC, EM, CP and HM analysed the results. SyP gave technical support for cell culture experiments. RC, HM, AG, LV, GC, EA and StP wrote the paper. All authors contributed to the article and approved the submitted version.

## Funding

This study received funding from Novartis DREAMER grant. The funder was not involved in the study design, collection, analysis, interpretation of data, the writing of this article or the decision to submit it for publication.

## Conflict of Interest

The authors declare that the research was conducted in the absence of any commercial or financial relationships that could be construed as a potential conflict of interest.

## Publisher’s Note

All claims expressed in this article are solely those of the authors and do not necessarily represent those of their affiliated organizations, or those of the publisher, the editors and the reviewers. Any product that may be evaluated in this article, or claim that may be made by its manufacturer, is not guaranteed or endorsed by the publisher.

## Supplementary Material

Supplementary Figure 1 Increased YAP activation in RA FLS by the time spent on stiff substrate. **(A)** Schematic representation of RA FLS culture experiment. FLS were passed on soft support at passage (P) 4 or P5 or P6 following immunofluorescence (IF) or RT-qPCR analysis; time spent by FLS on stiff support was increased at each passage (P6>P5>P4). **(B)** Epifluorescence representative images of YAP (IF technique, red), on RA FLS at x10 magnification at P4 and P5 (luminosity and contrast were enhanced identically for each images for clarity purpose); for all conditions, cells were cultured on soft (2 kPa) 96-well culture dishes coated with fibronectin. **(C)** Corresponding quantification of the percentage of cells with YAP nuclear localization for FLS from one RA patient (n=7 P4, n=8 P5), experiment was repeated for one other RA patient showing the same pattern. **(D, E)** qPCR quantification of ANKRD1 **(D)** or CYR61 **(E)** for FLS from one RA patient (n=6 P5 and n=4 P6) on soft (2 kPa) or stiff (2 GPa) support; results were normalized to HPRT. T-test and Mann-Whitney test *p < 0.05; **p < 0.01; ***p < 0.001. Data are presented as individual values with mean ± SD **(C)** or median and interquartile range **(D, E)**.

Supplementary Figure 2 YAP inhibition with siRNA on RA FLS. FLS from RA patient were grown on stiff (2 GPa) substrate coated with fibronectin for 72 hours. Following YAP or control siRNA transfection, FLS were treated with TNF and IL-17 for 48 hours. **(A, B)** RT-qPCR quantification of CYR61 **(A)** and Bcl2 **(B)**; results were normalized to HPRT. **(C)** Representative WB results of total YAP-TAZ, phospho-NF-κB p65 (serine536 = active form), total NF-κB p65, and β-actin. **(D-F)** WB quantification results for phospho-NF-κB / β-actin **(D)**, NF-κB / β-actin **(E)**, phospho-NF-κB / NF-κB **(D)** of four replicates for one arthritic patient. Mann-Whitney tests: *p < 0.05. Data are presented as individual values with median and interquartile range. TNF: 10 ng/ml, IL-17: 50 ng/ml.

Supplementary Figure 3 YAP inhibition decreases inflammatory markers in vivo and in vitro. Samples used for in vivo experiments came from the animal protocols described in Figure 4 (AIA rats). **(A)** Representative airyscan confocal tiled images for RORγt (immunofluorescence technique, red), counterstained with DAPI (blue), 10-µm thick cryosections of rat spleen, right image: corresponding cropped image of merged in the marginal zone (MZ). **(B-J)** RT-qPCR quantification in spleen and ankle as indicated for AIA rats experiments **(B-H)** and RA FLS **(I, J)**. Results were normalized to HPRT. Kruskal Wallis (KW) or ANOVA test with FDR post hoc tests corrected (q-value) for multiple comparisons. *q < 0.05; **q < 0.01; ***q < 0.001. Data are presented as individual values with mean ± SD **(B-D, F-H)** or median and interquartile range **(E, I-J)**. MZ, marginal zone; GC, germinal center; arrows: RORγt positive cells located in the marginal zone.

Supplementary Figure 4 Prevention of colitis in a mouse model by YAP/TAZ inhibition through similar mechanisms observed in arthritis. All animals received IP injection each day from day -1 to day 10; control (vehicle) or DSS mice (1.5% DSS in water) were injected with vehicle containing 10% DMSO, treated DSS mice were injected with 40 mg/kg/day of VP. **(A)** Disease activity index (DAI) represents the severity of the disease by evaluating percentage weight loss, general aspect of the mice like pain behavior and feces aspect; two-way ANOVA test with FDR post hoc test corrected (q-value) for multiple comparison; #: DSS vs. vehicle and *: DSS vs VP. **(B)** RT-qPCR quantification on intestinal tract; results are normalized to HPRT. **(C, D)** Representative brightfield tiled images at x200 magnification of 10-µm thick paraffin transverse sections of intestinal tract, H&E staining. **(C)** DSS, **(D)** DSS+VP; dotted line represents Peyer’s patches limit; black arrows: intestinal villi. **(E)** Corresponding quantification of Peyer’s patches area in DSS and DSS+VP group; results represent the mean of 3 sections per Peyer’s patches in duplicate for each mouse, Mann-Whitney test. **(F-J)** RT-qPCR quantification on intestinal tract for TNF **(F)**, IL6 **(G)** IL17 **(H)**, MMP13 **(I)**, MMP2 **(J)**, and tenascin-C **(K)**; results are normalized to HPRT. Kruskal Wallis (KW) or ANOVA test with FDR post hoc test corrected (q-value) for multiple comparisons. # or *q < 0.05; **q < 0.01; ***q < 0.001. Data are presented as individual values with median and interquartile range **(B)** or mean ± SD **(A, E, F-K)**.
