# Supplementary figures and images for: YAP/TAZ: Key Players for Rheumatoid Arthritis Severity by Driving Fibroblast Like Synoviocytes Phenotype and Fibro-Inflammatory Response
Source: Front Immunol. 2021 Dec 9;12:791907. doi: 10.3389/fimmu.2021.791907 (PMC8695934; doi:10.3389/fimmu.2021.791907)

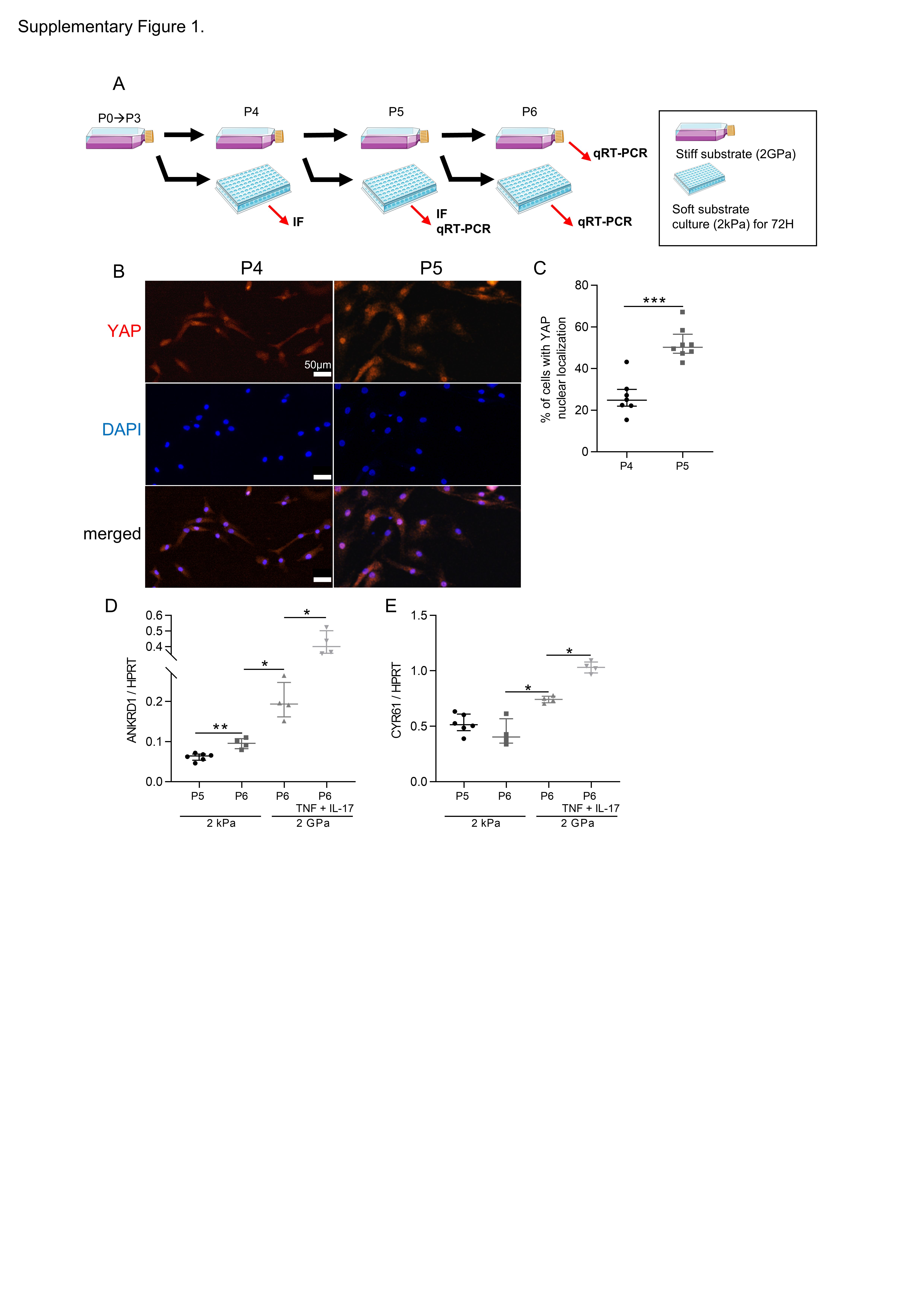

Supplement: Supplementary Figure 1 — Increased YAP activation in RA FLS by the time spent on stiff substrate. (A) Schematic representation of RA FLS culture experiment. FLS were passed on soft support at passage (P) 4 or P5 or P6 following immunofluorescence (IF) or RT-qPCR analysis; time spent by FLS on stiff support was increased at each passage (P6>P5>P4). (B) Epifluorescence representative images of YAP (IF technique, red), on RA FLS at x10 magnification at P4 and P5 (luminosity and contrast were enhanced identically for each images for clarity purpose); for all conditions, cells were cultured on soft (2 kPa) 96-well culture dishes coated with fibronectin. (C) Corresponding quantification of the percentage of cells with YAP nuclear localization for FLS from one RA patient (n=7 P4, n=8 P5), experiment was repeated for one other RA patient showing the same pattern. (D, E) qPCR quantification of ANKRD1 (D) or CYR61 (E) for FLS from one RA patient (n=6 P5 and n=4 P6) on soft (2 kPa) or stiff (2 GPa) support; results were normalized to HPRT. T-test and Mann-Whitney test *p < 0.05; **p < 0.01; ***p < 0.001. Data are presented as individual values with mean ± SD (C) or median and interquartile range (D, E). [file Image_1.tif]

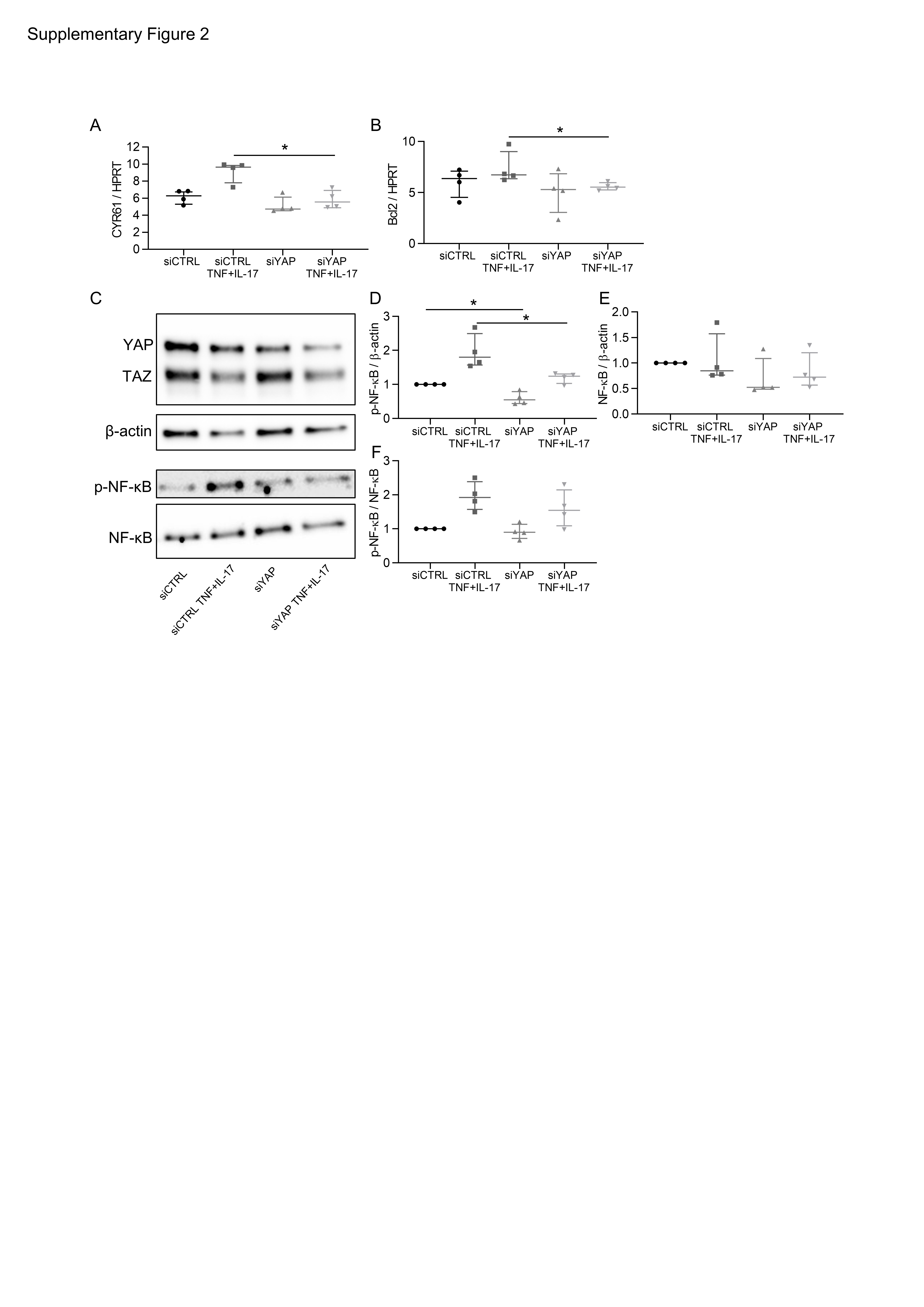

Supplement: Supplementary Figure 2 — YAP inhibition with siRNA on RA FLS. FLS from RA patient were grown on stiff (2 GPa) substrate coated with fibronectin for 72 hours. Following YAP or control siRNA transfection, FLS were treated with TNF and IL-17 for 48 hours. (A, B) RT-qPCR quantification of CYR61 (A) and Bcl2 (B); results were normalized to HPRT. (C) Representative WB results of total YAP-TAZ, phospho-NF-κB p65 (serine536 = active form), total NF-κB p65, and β-actin. (D-F) WB quantification results for phospho-NF-κB / β-actin (D), NF-κB / β-actin (E), phospho-NF-κB / NF-κB (D) of four replicates for one arthritic patient. Mann-Whitney tests: *p < 0.05. Data are presented as individual values with median and interquartile range. TNF: 10 ng/ml, IL-17: 50 ng/ml. [file Image_2.tif]

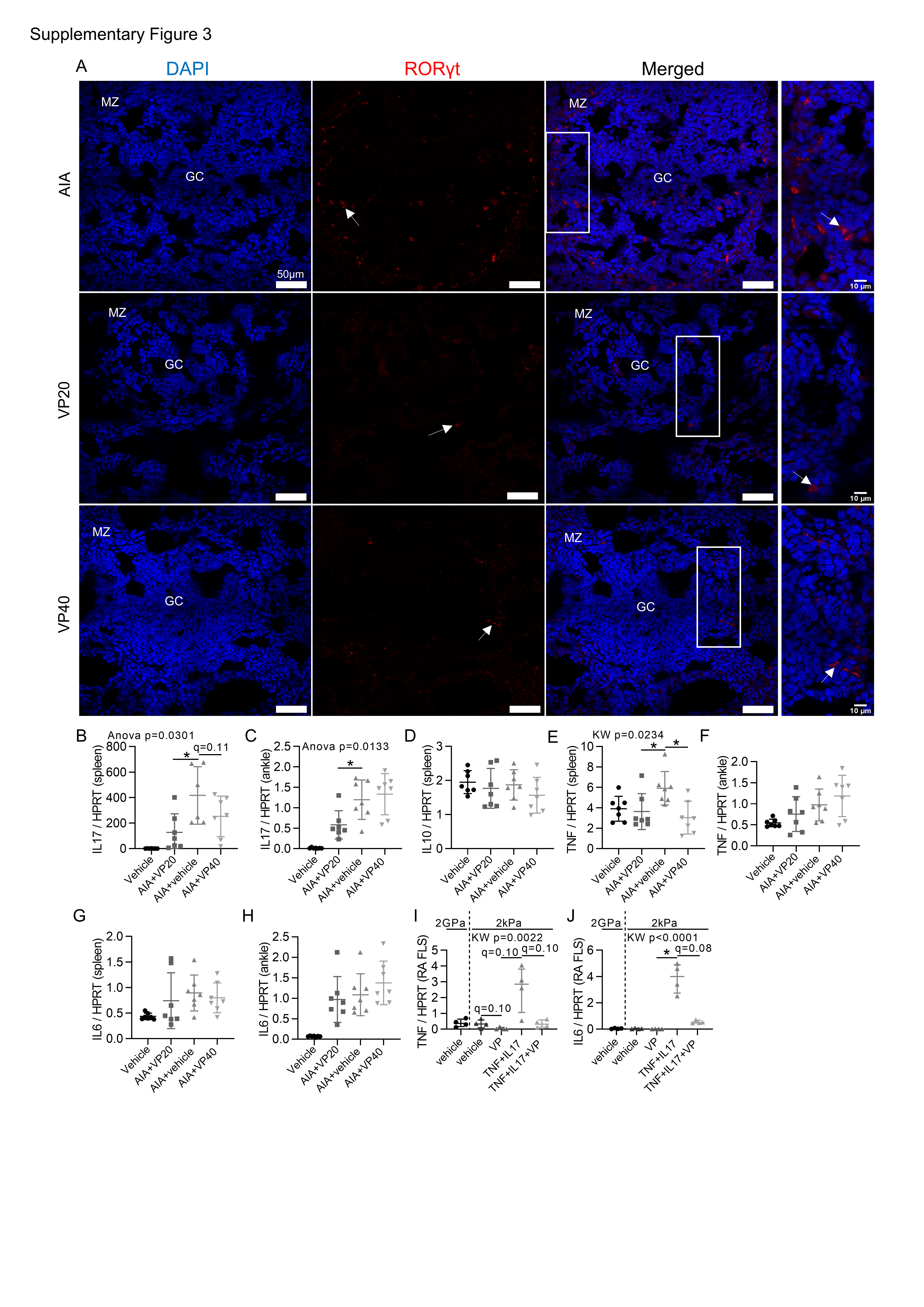

Supplement: Supplementary Figure 3 — YAP inhibition decreases inflammatory markers in vivo and in vitro. Samples used for in vivo experiments came from the animal protocols described in Figure 4 (AIA rats). (A) Representative airyscan confocal tiled images for RORγt (immunofluorescence technique, red), counterstained with DAPI (blue), 10-µm thick cryosections of rat spleen, right image: corresponding cropped image of merged in the marginal zone (MZ). (B-J) RT-qPCR quantification in spleen and ankle as indicated for AIA rats experiments (B-H) and RA FLS (I, J). Results were normalized to HPRT. Kruskal Wallis (KW) or ANOVA test with FDR post hoc tests corrected (q-value) for multiple comparisons. *q < 0.05; **q < 0.01; ***q < 0.001. Data are presented as individual values with mean ± SD (B-D, F-H) or median and interquartile range (E, I-J). MZ, marginal zone; GC, germinal center; arrows: RORγt positive cells located in the marginal zone. [file Image_3.tif]

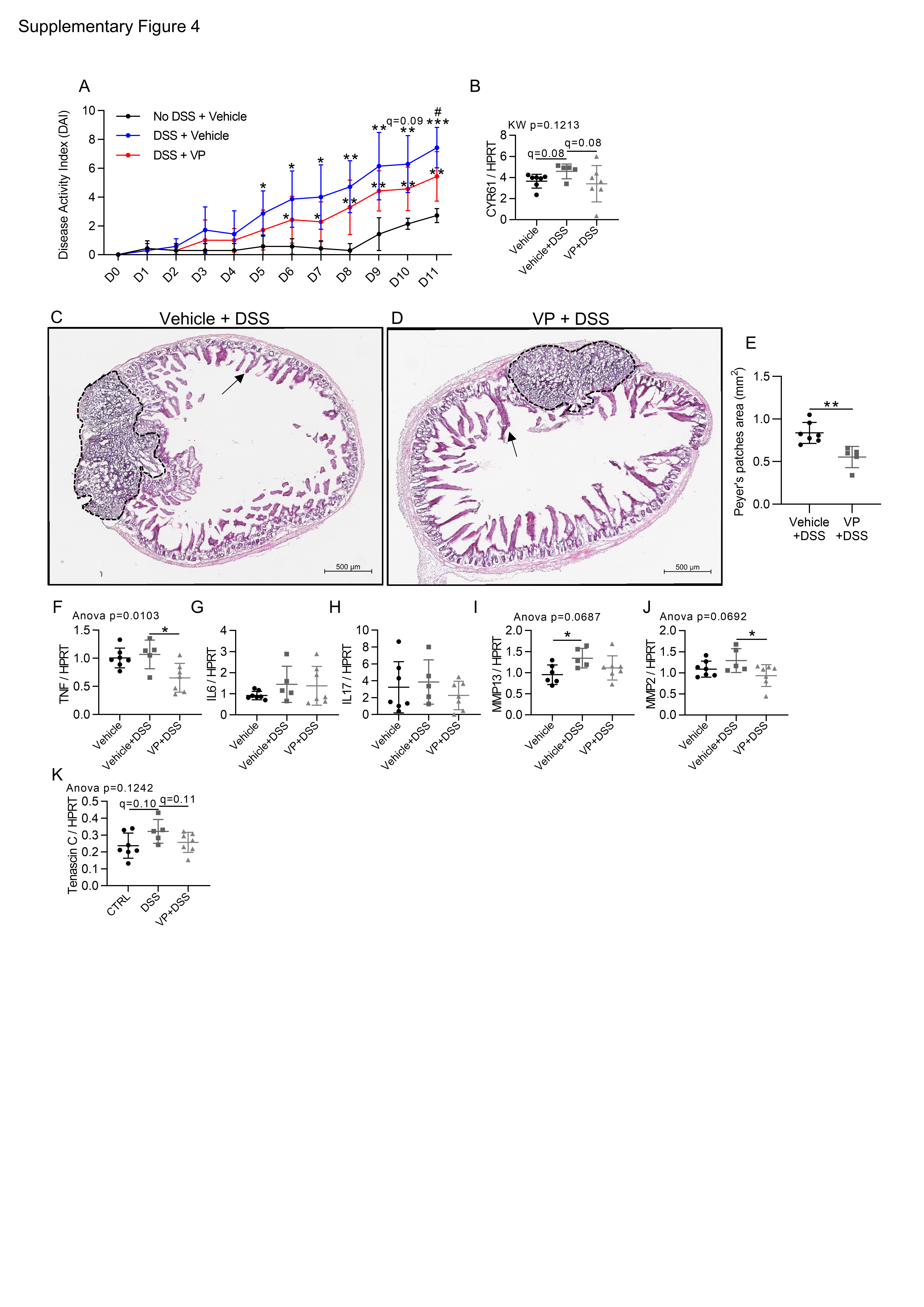

Supplement: Supplementary Figure 4 — Prevention of colitis in a mouse model by YAP/TAZ inhibition through similar mechanisms observed in arthritis. All animals received IP injection each day from day -1 to day 10; control (vehicle) or DSS mice (1.5% DSS in water) were injected with vehicle containing 10% DMSO, treated DSS mice were injected with 40 mg/kg/day of VP. (A) Disease activity index (DAI) represents the severity of the disease by evaluating percentage weight loss, general aspect of the mice like pain behavior and feces aspect; two-way ANOVA test with FDR post hoc test corrected (q-value) for multiple comparison; #: DSS vs. vehicle and *: DSS vs VP. (B) RT-qPCR quantification on intestinal tract; results are normalized to HPRT. (C, D) Representative brightfield tiled images at x200 magnification of 10-µm thick paraffin transverse sections of intestinal tract, H&E staining. (C) DSS, (D) DSS+VP; dotted line represents Peyer’s patches limit; black arrows: intestinal villi. (E) Corresponding quantification of Peyer’s patches area in DSS and DSS+VP group; results represent the mean of 3 sections per Peyer’s patches in duplicate for each mouse, Mann-Whitney test. (F-J) RT-qPCR quantification on intestinal tract for TNF (F), IL6 (G) IL17 (H), MMP13 (I), MMP2 (J), and tenascin-C (K); results are normalized to HPRT. Kruskal Wallis (KW) or ANOVA test with FDR post hoc test corrected (q-value) for multiple comparisons. # or *q < 0.05; **q < 0.01; ***q < 0.001. Data are presented as individual values with median and interquartile range (B) or mean ± SD (A, E, F-K). [file Image_4.tif]
